# Local Prefrontal Cortex TMS-Induced Reactivity Is Related to Working Memory and Reasoning in Middle-Aged Adults

**DOI:** 10.3389/fpsyg.2022.813444

**Published:** 2022-02-10

**Authors:** María Redondo-Camós, Gabriele Cattaneo, Ruben Perellón-Alfonso, Vanessa Alviarez-Schulze, Timothy P. Morris, Javier Solana-Sanchez, Goretti España-Irla, Selma Delgado-Gallén, Catherine Pachón-García, Sergiu Albu, Henrik Zetterberg, Josep M. Tormos, Alvaro Pascual-Leone, David Bartres-Faz

**Affiliations:** ^1^Institut Guttmann, Institut Universitari de Neurorehabilitació adscrit a la Universitat Autónoma de Barcelona, Barcelona, Spain; ^2^Departament de Medicina, Facultat de Medicina, Universitat Autònoma de Barcelona, Barcelona, Spain; ^3^Fundació Institut d'Investigació en Ciències de la Salut Germans Trias i Pujol, Barcelona, Spain; ^4^Institut d'Investigacions Biomèdiques August Pi i Sunyer (IDIBAPS), Barcelona, Spain; ^5^Departament de Medicina, Facultat de Medicina i Ciències de la Salut, i Institut de Neurociències, Universitat de Barcelona, Barcelona, Spain; ^6^Departamento de Ciencias del Comportamiento, Escuela de Psicología, Universidad Metropolitana, Caracas, Venezuela; ^7^Center for Cognitive and Brain Health, Department of Psychology, Northeastern University, Boston, MA, United States; ^8^Department of Psychiatry and Neurochemistry, Institute of Neuroscience and Physiology, the Sahlgrenska Academy at the University of Gothenburg, Mölndal, Sweden; ^9^Clinical Neurochemistry Laboratory, Sahlgrenska University Hospital, Mölndal, Sweden; ^10^Department of Neurodegenerative Disease, University College London Institute of Neurology, London, United Kingdom; ^11^UK Dementia Research Institute, University College London, London, United Kingdom; ^12^Hong Kong Center for Neurodegenerative Diseases, Hong Kong, China; ^13^Hinda and Arthur Marcus Institute for Aging Research and Deanna and Sidney Wolk Center for Memory Health, Hebrew SeniorLife, Boston, MA, United States; ^14^Department of Neurology, Harvard Medical School, Boston, MA, United States

**Keywords:** transcranial magnetic stimulation (TMS), Electroencephalography, TMS-EEG, cortical reactivity, prefrontal cortex (PFC), cognition

## Abstract

**Introduction:**

The prefrontal cortex (PFC) plays a crucial role in cognition, particularly in executive functions. Cortical reactivity measured with Transcranial Magnetic Stimulation combined with Electroencephalography (TMS-EEG) is altered in pathological conditions, and it may also be a marker of cognitive status in middle-aged adults. In this study, we investigated the associations between cognitive measures and TMS evoked EEG reactivity and explored whether the effects of this relationship were related to neurofilament light chain levels (NfL), a marker of neuroaxonal damage.

**Methods:**

Fifty two healthy middle-aged adults (41–65 years) from the Barcelona Brain Health Initiative cohort underwent TMS-EEG, a comprehensive neuropsychological assessment, and a blood test for NfL levels. Global and Local Mean-Field Power (GMFP/LMFP), two measures of cortical reactivity, were quantified after left prefrontal cortex (L-PFC) stimulation, and cognition was set as the outcome of the regression analysis. The left inferior parietal lobe (L-IPL) was used as a control stimulation condition.

**Results:**

Local reactivity was significantly associated with working memory and reasoning only after L-PFC stimulation. No associations were found between NfL and cognition. These specific associations were independent of the status of neuroaxonal damage indexed by the NfL biomarker and remained after adjusting for age, biological sex, and education.

**Conclusion:**

Our results demonstrate that TMS evoked EEG reactivity at the L-PFC, but not the L-IPL, is related to the cognitive status of middle-aged individuals and independent of NfL levels, and may become a valuable biomarker of frontal lobe-associated cognitive function.

## Introduction

Cognitive functioning refers to a set of multiple mental abilities that involve the overall dynamics of information processing of stimuli (acquisition, coding, storage, retrieval, thinking, and decision making) to generate an adequate response (motor or verbal) to the environment (Lezak et al., 2004). The characterization of cognitive performance and cognitive profiles is typically carried out through specific domains referring to different processes and abilities within the global term “cognition” (Harvey, 2019), such as attention, episodic memory, working memory, reasoning, fluid intelligence, language, cognitive flexibility, visuospatial skills, and processing speed.

These cognitive domains have been related to specific anatomic areas and brain networks (Wu et al., 2020), and amongst them, the prefrontal cortex (PFC) plays a central role. The PFC is connected to almost all sensory, motor, neocortical and subcortical structures and is often implicated in “top-down” modulation of cognitive functions (Miller, 2000). In a hierarchical model of the neurophysiology of the cortex, the PFC constitutes the highest area of cortical representations (as opposed to lower cortical structures such as sensory and motor areas) dedicated to the integration and execution of higher-order executive functions (Fuster, 2001; Breukelaar et al., 2018). As these functions are significantly affected during aging, the role of the PFC has been extensively studied, and it has been shown that greater PFC activity is associated with better cognition (Eyler et al., 2011; Fernandez-Ruiz et al., 2018) and that preservation of PFC activity contributes to the maintenance of cognitive abilities (Morcom and Henson, 2018; Vidal-Piñeiro et al., 2019).

Beyond correlational evidence from brain-behavior investigations, direct experimental data using repetitive transcranial magnetic stimulation (rTMS) to modulate older adults' PFC function demonstrated that high-frequency rTMS delivered over the bilateral PFC can enhance cognitive functioning (e.g. Solé-Padullés et al., 2006; Cui et al., 2020). If interpreted in the framework of theoretical models of cognitive aging, this enhancement may be due to the promotion of compensatory mechanisms by rTMS (Cabeza et al., 2018).

Given the role of the PFC in cognition and its relevance in healthy aging, the characterization of its neurophysiological activity in middle-aged adults, often understudied as compared with other age brackets (Willis et al., 2010; Lachman, 2015), could represent a valuable biomarker predictive of cognitive decline in older adults (McGinnis et al., 2011). Furthermore, research in middle-aged populations is relevant because changes in brain function can occur decades prior to the onset of clinically measurable symptoms (Beason-Held et al., 2013).

Transcranial Magnetic Stimulation combined with Electroencephalography (TMS-EEG) is a non-invasive approach that allows the study of cortical reactivity via the perturbation of a cortical site and registration of the activity spread throughout the brain (Ilmoniemi and Kičić, 2010; Hallett et al., 2017). The spatiotemporal analysis of this reactivity and propagation allowed previous studies to explore functional network integrity in healthy and clinical populations (Pascual-Leone et al., 2011; Tremblay et al., 2019; Ozdemir et al., 2020).

Cortical reactivity has been defined as the relationship between the strength of the stimulus and the subsequent response (Komssi and Kähkönen, 2006). It is relevant because optimal excitatory and inhibitory cortical balance is needed for the correct functioning of the brain, connectivity between cortical regions, and cognitive functioning (Dehghani et al., 2016). Local and Global Mean-Field Power (GMFP/LMFP) reflect TMS-evoked brain reactivity on a specific subgroup of electrodes or throughout the entire brain, respectively (Lehmann and Skrandies, 1980; Komssi and Kähkönen, 2006; Romero Lauro et al., 2014). Both measures can reflect the electrical field distribution on the scalp (Skrandies, 1990). They could be valuable for measuring local reactivity directly on the stimulated region and reflecting its distribution to other brain areas (Lehmann and Skrandies, 1980). The neurophysiological effect of non-invasive protocols (repetitive TMS or Transcranial Direct Current Stimulation) has been studied through these measures in healthy adults (Casarotto et al., 2010; Romero Lauro et al., 2014; Pisoni et al., 2018; Ozdemir et al., 2021) and earlier research showed how these measures were directly related to pathological conditions, like depression (Voineskos et al., 2019). However, the relation between GMFP/LMFP and cognition in a healthy middle-aged population has not been studied before.

The goal of this study was to explore the relationship between cognition and local and global cortical reactivity after PFC stimulation using TMS-EEG in healthy middle-aged adults. Moreover, we investigated whether the effects are independent of a general measure of neuroaxonal damage and neurodegeneration as derived from plasma neurofilament light chain (NfL; Gisslén et al., 2016), a biomarker that has been associated with cognitive decline amongst elderly adults in the preclinical phase of Alzheimer's disease (AD) (Hu et al., 2019) and potentially in healthy middle-aged adults (Beydoun et al., 2021).

## Materials and Methods

### Subjects and Study Design

Fifty two middle-aged adults (36 male) between 41 and 65 years (*M* = 54, *SD* = 6.85) were recruited as part of the Barcelona Brain Health Initiative (Cattaneo et al., 2018). They underwent a TMS-EEG session, neuropsychological testing, a medical assessment with blood sample collection, and structural Magnetic Resonance Imaging (MRI). Participants were excluded during the medical visit if they had any neurological or neuropsychiatric disorders or used medications that could affect brain excitability or cognitive functions. Further exclusion criteria included contraindications for TMS (Rossini et al., 2015; Rossi et al., 2021) or MRI. All participants gave written informed consent, and the local ethics committee (Comité d'Ètica i Investigació Clínica de la Unió Catalana d'Hospitals) approved the study protocol, which conformed to the Declaration of Helsinki for research involving human subjects.

### Neuropsychological Assessment

The neuropsychological assessment consisted of paper and pencil tests administered by two licensed neuropsychologists (VA, CP). The testing session lasted ~90 min and included 14 validated gold-standard instruments (see Cattaneo et al., 2018): Rey Auditory Verbal Learning Test (RAVLT) (Schmidt, 1996), Digit-Span Forward and Backward, Corsi block tapping test, Letter-Number Sequencing test (Peña-Casanova et al., 2012), Trail Making Test A and B (TMT) (Reitan and Wolfson, 1985; Peña-Casanova et al., 2012), Matrix Reasoning and Block design, the Digit symbol task, and the Cancellation test (Wechsler, 2012).

Raw scores of each test were transformed into z-scores and, similar to our previous reports (Vidal-Piñeiro et al., 2014; España-Irla et al., 2021), were grouped into five cognitive domains: episodic memory (RAVLT immediate recall, delayed recall, and recognition; Digit-span forward; Corsi block tapping), working memory (Digit-span backward; Letter-number sequencing), reasoning (Matrix and Block design WAIS-IV), flexibility (TMT B and B-A), and processing speed (TMT A; Digit symbol test; Cancellation test).

### TMS Protocol

Participants were asked to sit in a comfortable armchair, look at a fixation cross placed at a 1.5 m distance, stay still, and keep their eyes open. The coil was placed tangentially over the scalp roughly at a 45-degree angle (relative to the mid-sagittal plane), resulting in a posterior-to-anterior current flow. A frameless stereotactic neuronavigation system (Brainsight, Rogue Research Inc., Montreal, QC Canada) was used with the subject's T1 weighted structural MRI (obtained from a 3T Siemens Magnetom Prisma) to ensure accurate targeting of the stimulation sites throughout the session.

Participant's Resting Motor Threshold (RMT) was assessed at the motor hotspot (M1) of the dominant hemisphere following the recommendations from the International Federation for Clinical Neurophysiology (Rossini et al., 2015; Rossi et al., 2021). Briefly, RMT was defined as the lowest stimulation intensity required to produce motor-evoked potentials (MEPs) of ≥ 50 μV in the relaxed first dorsal interosseous muscle (FDI) in five out of 10 trials. MEPs were measured using surface electromyography (EMG) with electrodes placed in a belly-tendon montage and the ground electrode on the ulnar styloid process and connected to a Biopac EMG100C amplifier (BIOPAC Systems Inc., California, USA). Handedness was assessed by the Edinburgh handedness questionnaire (Oldfield, 1971; Veale, 2014).

TMS was applied using a Medtronic Magpro X100 stimulator through a Cool-B65 figure-of-eight coil. One hundred and twenty biphasic single pulses were applied at 120% of RMT at random intervals between 3 and 6 s. Also, stimulation was applied over two target locations: the left prefrontal cortex (L-PFC) and a control target, the Inferior Parietal Lobule (L-IPL). The cohort had two different target procedures, based on the anatomy of each subject or using a cortical functional parcellation (Yeo et al., 2011) (See Supplementary Material for details on targeting procedure).

Due to time constraints during the experimental sessions, 77% of the individuals completed L-PFC stimulation (a total of 40 participants) and 67% L-IPL (35 participants), with 44% of them completing both conditions (23 participants). The complete TMS procedure lasted 2 h.

### EEG Recordings

The EEG equipment used to record EEG responses to TMS was made up of a TMS-compatible EEG amplifier (ActiChamp system, Brain Products, GmbH, Munich, Germany) attached to 64 active electrodes (ActiCAP slim, Brain Products, GmbH, Munich, Germany), following the 10–20 international system for electrode montage. The ground was placed at the Fpz electrode site, and the signal was referenced to the AFz electrode. Electrode impedances were kept below 5 kΩ during the recording, and a continuous signal was collected, filtered DC to 500 Hz, and digitized at a sampling rate of 1,000 Hz. Besides wearing earplugs to protect from the “click” of the TMS pulse, subjects listened to white noise during stimulation to dampen the auditory evoked potential. The volume of the white noise was individually adjusted to each subjects' tolerance, and it was played through an active noise-canceling inserted earphone (Beoplay E4, Bang&Olufsen, Denmark).

### EEG Preprocessing

The EEG signal was first preprocessed offline with custom MATLAB scripts (R2020b, The MathWorks Inc., Natick, Massachusetts) that incorporate function from the EEGLAB toolbox (Delorme and Makeig, 2004) and TESA plugin (Rogasch et al., 2017). The EEG signal was epoched around the TMS pulse (−1,000 to +2,000 ms) and baseline corrected (−900 to −100 ms). Excessively noisy channels were removed, but no more than three channels had to be discarded for any subject. Data was zero-padded between −2 and +14 ms around the TMS pulse to remove the early TMS pulse artifact. Epochs were inspected visually, and excessively noisy epochs were removed (*M* = 19, *SD* = 7). A two-step fast Independent Component Analysis (fICA) was conducted. The first fICA, was performed with Principal Component Analysis dimension reduced to 40 to minimize overfitting and was used to remove the decay artifact, typically 2 components were removed for each subject. Before the second round of fICA, the zero-padded TMS pulse was linearly interpolated, Butterworth band-pass (1 and 100 Hz) and notch (48 and 52 Hz) filters were applied, and data were re-referenced to the average reference. The second round of fICA was used to remove any remaining artifacts, including eye blinks, lateral eye movement, muscle, TMS-evoked muscle, electrode noise, and auditory evoked potentials (*M* = 28, *SD* = 3; a range of 21–31 out of 38). Finally, initially discarded channels were spline interpolated.

### Cortical Reactivity TMS-EEG Measures

Global Mean-Field Power (GMFP) and Local Mean-Field Power (LMFP) were used to quantify overall and local brain reactivity measures, respectively.

Mean Field-Power (MFP) was calculated for both measures using the following formula:


$$\text{MF}P\;\left(t\right)=\sqrt{\frac{\left[\sum_{i}^{k}\;{\left(V_{i}\left(t\right)-V_{mean\;}(t)\right)}^{2}\right]}{K}}$$

where “t” is time, “V” is the voltage in the channel “I,” “K” is the number of channels, and “Vmean” the mean of the voltage across electrodes (Lehmann and Skrandies, 1980; Esser et al., 2006).

All EEG electrodes were used to compute GMFP, whereas, for LMFP, a subset of electrodes was chosen for L-PFC (FC1, FC3, FC5, F1, F3, F5) and L-IPL (CP1, CP3, CP5, P1, P3, P5) (Ozdemir et al., 2020). LMFP was used to calculate the local reactivity of the stimulation target region.

For GMFP and LMFP, the area under the curve was calculated using trapezoidal integration within two-time windows, before and after the pulse (Baseline and Post-stimulation). Baseline refers to the activity before each pulse (−500 to −3 ms), while Post-stimulation activity includes data between 15 and 400 ms after the TMS pulse. This time window has been selected to minimize the TMS artifact's impact and capture the entirety of the TMS evoked brain response (Fuggetta et al., 2005; Van Der Werf et al., 2006). This time window has been used in recent and similar research (Ozdemir et al., 2020; Rocchi et al., 2020; Vallesi et al., 2021). Furthermore, baseline data was used to normalize the activity post-stimulation, subtracting it from the activity post-TMS.

### NfL Measurement

We collected blood samples using EDTA tubes during the medical assessment, and plasma was aliquoted and stored in a refrigerator at −80°C in a biobank facility following standard procedures usually employed for clinical purposes. Plasma NfL concentration was measured using the Single-molecule array (Simoa) NF-light Advantage Kit on an HD-X instrument as described by the kit manufacturer (Quanterix, Billerica, MA). The limit of quantification was 2.7 pg/mL, and the limit of detection was 0.3 pg/mL. For the quality control (QC) sample with an 11.2 pg/mL concentration, repeatability was 3.6%, and intermediate precision was 5.0%. For a QC sample with a 115 pg/mL concentration, repeatability was 5.3%, and intermediate precision was 6.8%. The measurements were performed at the Clinical Neurochemistry Laboratory at the University of Gothenburg by board-certified laboratory technicians who were blinded to clinical data.

### Statistical Analysis

All statistical analyses were performed in SPSS version 22.0 (Statistical Package for Social Sciences, Chicago, IL, USA).

First, to explore changes between local and global reactivity before and after stimulation, we ran a repeated-measures ANOVA using the variable “Time” (Baseline and Post-stimulation) and “Mean-Field Type” (LMFP and GMFP) as within-subject factors.

Then, to investigate the relation between TMS reactivity, at local and global levels, and cognition, we ran multivariate multiple regressions for each stimulation site (PFC, IPL). L-IPL was used as a control condition to validate if our associations with cognition in L-PFC results were specific to this target. Models were run using cognitive composite scores as dependent variables (episodic memory, working memory, reasoning, flexibility, and processing speed) and Mean-Field Type, targeting method, NfL levels, age, biological sex, and years of education as predictors. Also, we run the same models without using covariates (targeting method, NfL levels, age, biological sex, and years of education).

Finally, to specifically explore the associations between NfL level and cognition, we first performed a bivariate correlation, and then to see the effect of age, we ran a partial correlation controlled by this variable.

## Results

All subjects were right-handed and tolerated well the experimental procedures, and no adverse events were reported. Descriptive statistics of age, educational level, RMT, and plasma NfL levels are presented in Table 1, while cognitive scores are in the Supplementary Material (Supplementary Table 1).

**Table 1:** Demographic Variables, RMT and NfL plasma values (*n* = 52).

	**Min**	**Max**	**Mean**	**SD**
Age	41	65	53.96	6.85
Years of Education	8	28	18.40	3.83
RMT	43	82	59.77	8.76
NfL levels (pg/mL)	4.09	29.8	12.22	5.35

### TMS Cortical Reactivity Changes

#### L-PFC

**Figure 1:**
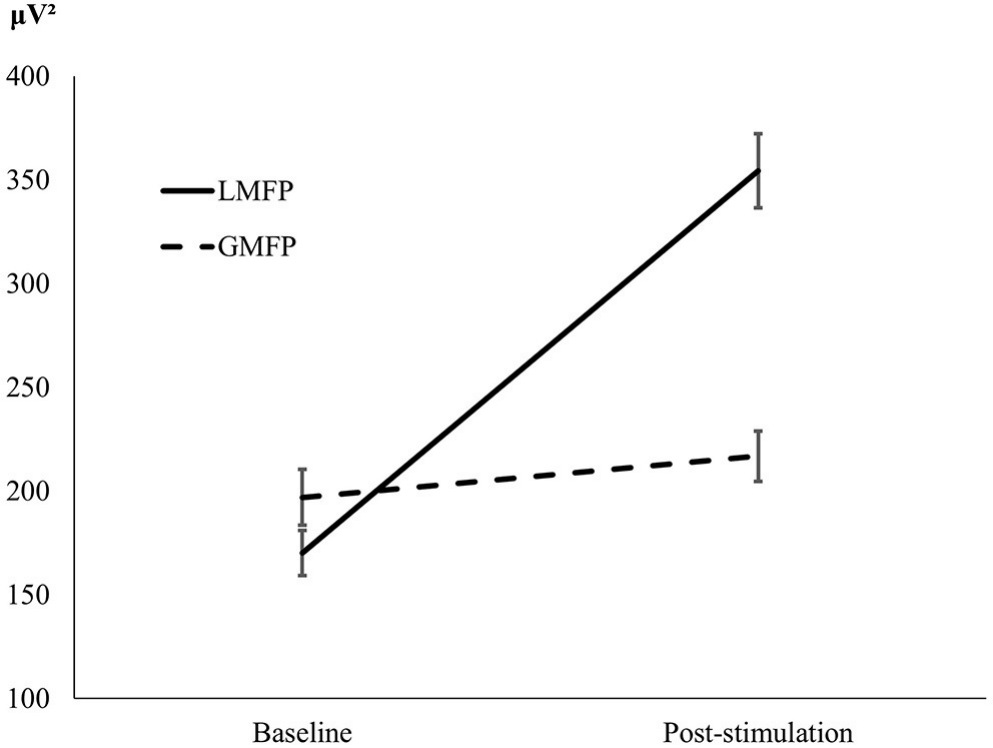
L-PFC. Graphical representation of local (LMFP) and global (GMFP) reactivity, at baseline and after stimulation.

Repeated measures ANOVA showed a main effect of Time [*F*_(1, 39)_ = 54.05, *p* < 0.001], indicating difference between pre and post stimulation, and a main effect of the Mean-Field Type [*F*_(1, 39)_ = 69.02, *p* < 0.001], indicating that local reactivity was greater (*M* = 262.32; *SD* = 13.90) than global cortical reactivity (*M* = 206.91; *SD* = 9.54). The significant interaction between Time and Mean-Field Type [*F*_(1, 39)_ = 145.97, *p* < 0.001] indicated a difference in the effects of stimulation between “local” and “global” conditions. While LMFP showed significant differences between baseline and post-TMS [*t*_(39)_ = 13.56, *p* < 0.001, *d* = 2.14] GMFP did not [*t*_(39)_ = 1.16, *p* = 0.253, *d* = 0.18; see Figure 1].

#### L-IPL

**Figure 2:**
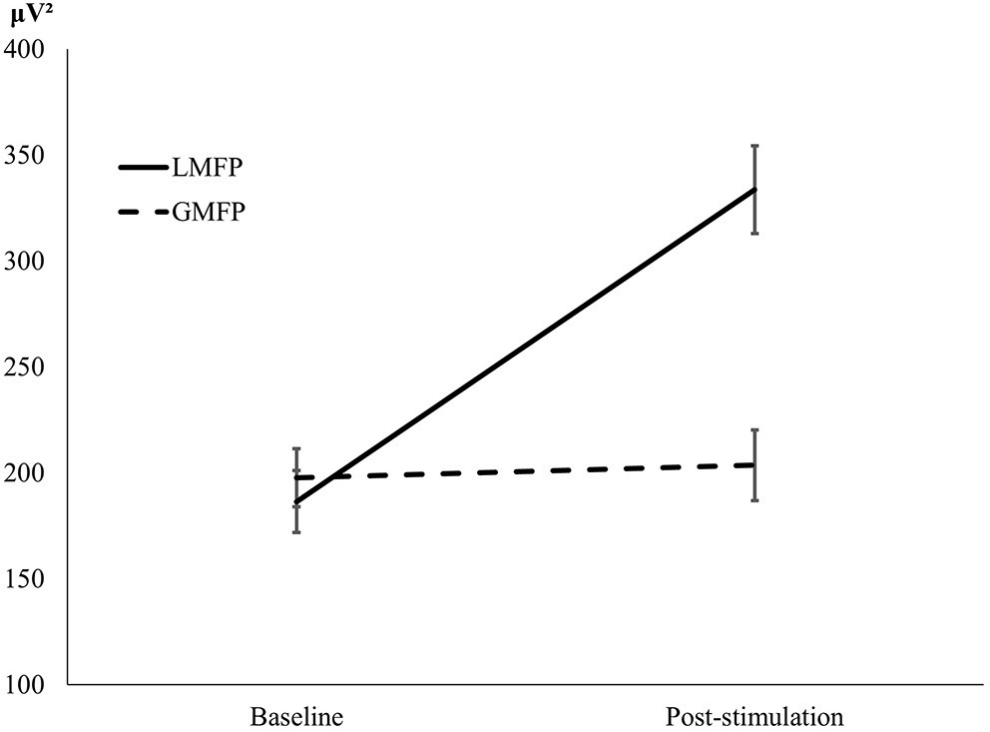
L-IPL. Graphical representation of LFMP and GFMP at baseline and after stimulation.

**Figure 3:**
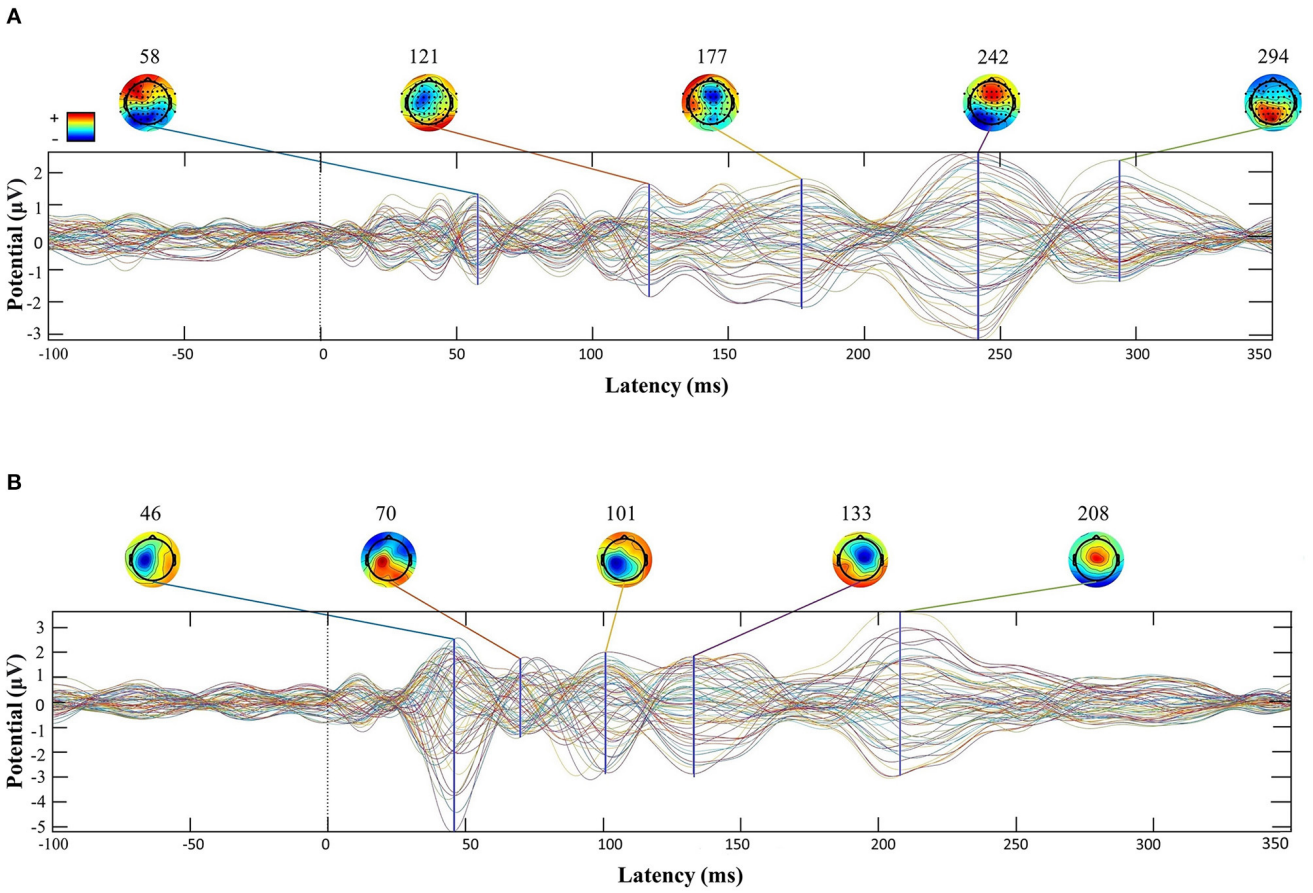
Butterfly plots of a subject's TMS-EEG responses after PFC **(A)** and IPL **(B)** stimulation. Each figure time-series are plotted −100 to +400 ms around the TMS pulse.

Repeated measures ANOVA also showed a main effect of Time [*F*_(1, 34)_ = 20.01, *p* < 0.001], indicating difference between pre and post stimulation, and a main effect of the Mean-Field Type [*F*_(1, 34)_ = 89.38, *p* < 0.001] showing that local reactivity was greater (*M* = 260.10; *SD* = 15.78) than global cortical reactivity (*M* = 200.63; *SD* = 12.02). The significant interaction between Time and Mean-Field Type [*F*_(1, 34)_ =143.96, *p* < 0.001] suggested difference in the effects of stimulation between “local” and “global” conditions. Similarly to the L-PFC analysis, while LMFP showed significative differences between baseline and post-TMS [*t*_(34)_ = 8.55, *p* < 0.001, *d* = 1.44] GMFP did not [t_(34)_ = 0.311, *p* = 0.758, *d* = 0.05; see Figure 2]. Also, an example of TMS-EEG responses after a PFC and IPL pulse is presented in Figure 3.

### Cortical Reactivity and Cognitive Functions Associations

#### L-PFC

**Figure 4:**
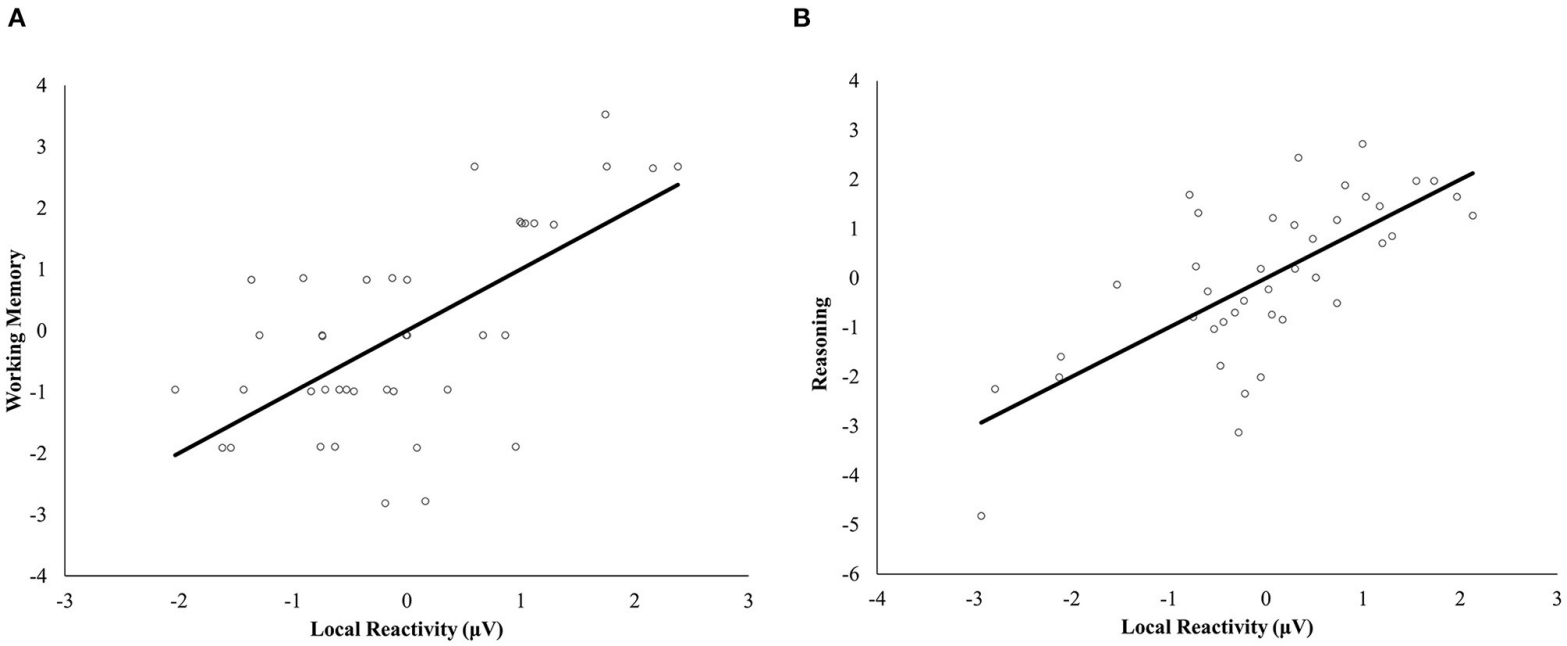
Multiple regression scatterplots between local cortical reactivity after PFC stimulation and working memory **(A)** and reasoning **(B)** after controlling for targeting method, NfL levels, age, biological sex, and years of education. Z-scores were used on the Y-axis and unstandardized Predicted Values on the X-axis (μV).

Age and local reactivity resulted in statistically significant results for the multivariate regression analysis [respectively *F*_(5, 28)_ = 3.28, *p* < 0.019, Wilks' Λ = 0.631, partial η2 = 0.369; *F*_(5, 28)_ = 2.91, *p* = 0.031, Wilks' Λ = 0.658, partial η2 = 0.342; see Supplementary Table 2]. The analysis revealed a significant positive association between working memory and local reactivity to stimulation of the L-PFC [*F*_(1, 32)_ = 5.01, *p* = 0.032, partial η2 = 0.135], as well as an association between reasoning and both age [*F*_(1, 32)_ = 6.76, *p* = 0.014, partial η2= 0.174] and local reactivity to L-PFC stimulation [*F*_(1, 32)_ = 4.70, *p* = 0.038, partial η2= 0.128] (see Figure 4). Episodic memory, processing speed, and flexibility were unrelated to the independent variables introduced in the model. Full model results including covariates can be found in Supplementary Table 3.

Conversely, no significant results were seen between cognition and cortical reactivity in models without covariates (see Supplementary Tables 4, 5).

#### L-IPL

For stimulation to left IPL, no statistically significant relations were found between any of the cognitive functions and either global or local TMS-EEG induced reactivity measures at baseline or post-stimulation. Model results including covariates are presented in Supplementary Tables 6, 7. Also, no significant results were seen between cognition and cortical reactivity in models without covariates (see Supplementary Tables 8, 9).

### NfL Levels and Cognitive Functions

We first ran a bivariate correlation between NfL levels and cognitive variables and found that it was significantly and inversely correlated to participants' cognitive performance in reasoning (*r* = −0.373, *p* = 0.006), processing speed *(r* = −0.338, *p* = 0.014), and cognitive flexibility (*r* = −0.293, *p* = 0.035; see Supplementary Table 10). However, when controlled for age, such associations disappeared, indicating that the age of participants largely drove the correlations. In addition, and as revealed by the multivariate regression analyses, NfL concentration was not significantly associated with cognitive status (see Supplementary Tables 3, 7).

## Discussion

In the present study, we explored the relationship between EEG reactivity to TMS of the PFC and IPL and cognition in healthy middle-aged adults, and the possible role of neuroaxonal damage measured by plasma NfL. Results indicate that local TMS-EEG reactivity after PFC stimulation is positively associated with executive functions, specifically working memory and reasoning. However, after IPL stimulation, cortical reactivity was not related to cognitive function. Finally, it was shown that neuroaxonal damage measured by NfL did not play a role in these associations. Despite that, a significant effect was seen when we correlated directly NfL level and cognitive functions, which disappeared when we controlled by age.

The fact that the relation between TMS-EEG reactivity and cognitive were limited to the local response following PFC stimulation, and not globally or when stimulating the L-IPL, is consistent with the specific PFC role in the top-down regulation of higher-order cognitive control (Miller, 2000). These results indicate that individual differences in local cortical reactivity to TMS of the PFC could be a practical, specific, sensitive, and simple biomarker to assess cognitive functioning, independently of the global brain and axonal degeneration.

Cortical reactivity has been previously related to factors such as alcohol or medication intake (Kähkönen et al., 2003; Khedr et al., 2020) and various TMS parameters (Casula et al., 2018). A rich literature has demonstrated that activity and connectivity between PFC and IPL are associated with cognition (Lückmann et al., 2014; Friedman and Robbins, 2021). In particular, whereas PFC has been more related to executive function and cognitive control (Friedman and Robbins, 2021), IPL has been associated with language and social cognition (Numssen et al., 2021). In line with our results, Ngetich et al. (2020) showed that after a continuous theta burst stimulation over L-PFC, there was a change in executive functions like working memory and decision making (Ngetich et al., 2020). Similarly, it has been shown that abnormal higher cortical excitability in the PFC in patients with AD than healthy controls was inversely associated with global cognition/executive functions (Joseph et al., 2021), confirming the relation between cognitive functions performance and PFC evoked activity.

The role of PFC in working memory has been extensively studied in the past decade with animals and humans, suggesting that PFC is strongly related to the cognitive process of maintaining available and select information for delayed responses (see Curtis and D'Esposito, 2003 for a review). It has been proposed that while the PFC is crucial to manipulate and select relevant information, a more posterior part of PFC (e.g., Brodmann area 8) is involved in mechanisms of maintenance (Rowe et al., 2000; Glahn et al., 2002). Also, complex reasoning tasks have been consistently associated with PFC activity and integrity. It has been proposed that PFC is strongly involved in logic processing (Santarnecchi et al., 2013), and specifically, its rostrolateral part is essential for relational integration and associations (Christoff et al., 2001; Krawczyk et al., 2011).

The association between cognition and PFC activity and connectivity is especially important in studying the maintenance of cognitive functioning in aging. Indeed it has been proposed that PFC activity could be related to the recruitment of compensatory mechanisms (Solé-Padullés et al., 2006; Höller-Wallscheid et al., 2017; Abellaneda-Pérez et al., 2019) that allow individuals to maintain cognition in the face of age-related brain changes.

Furthermore, NfL was shown to be negatively related to cognition, but this effect disappeared if controlled by the individual's age, and NfL level didn't have a significant influence on the identified relation between cortical reactivity and cognitive functions. NfL level is a marker of neuro-axonal damage in diseases such as Alzheimer's disease, Parkinson's disease, multiple sclerosis, or amyotrophic lateral sclerosis, where high concentrations of NfL have been reported (Gaetani et al., 2019; Dhiman et al., 2020). Recent studies (Khalil et al., 2020; Beydoun et al., 2021; Rübsamen et al., 2021) have explored in healthy individuals the relation between NfL levels, brain structures, and cognitive scores, suggesting that higher NfL levels could be associated with brain atrophy, and in consequence worse cognition. Our study sample was limited to middle-aged, cognitively-unimpaired adults, and the fact that the level of NfL didn't influence the reactivity/cognition prediction could be because of collinearity between age and NfL level, or that most past results have focused on older adults or various patient populations. More studies in healthy middle-aged adults are needed to determine the significance and potential clinical utility of NfL plasma levels (Beydoun et al., 2021).

To conclude, our results indicate that cortical reactivity of L-PFC as characterized by TMS-EEG is related to cognition in middle-aged adults regardless of neuroaxonal damage (indicated by NfL), age, biological sex, and education. This TMS-EEG metric may represent a valuable and independent biomarker for cognition.

As with all studies, the design of the current study is subject to limitations. First, we acknowledge that our sample size was small, but still, it was in line with other studies whose objective was to associate TMS measures with cognition. Second, our sample was characterized by highly educated individuals, and there was a high prevalence of men. Furthermore, our statistical analysis was done for each stimulation site separately because of missing data. Given the small sample sizes, multiple comparisons corrections were not applied to maintain statistical power and avoid strongly increasing the probability of type II errors. Hence, further studies are needed, including more participants with both PFC and IPL stimulation data to confirm these results. Finally, a layer of foam between the coil and the electrodes was not used in this research, and despite this preventive measure could add some distance, increasing the resting motor threshold and the effect varies between subjects (ter Braack et al., 2015), it could have been beneficial for the reduction of auditory evoked potentials in the EEG analysis. Future investigations with larger and more heterogeneous samples are necessary to validate the conclusions of our study, and it would be valuable to explore if changes in cortical reactivity, measured longitudinally, may be predictive of age-related changes in cognition.

## Data Availability Statement

The raw data supporting the conclusions of this article will be made available by the authors, without undue reservation.

## Ethics Statement

The studies involving human participants were reviewed and approved by Comité d'Ètica i Investigació Clínica de la Unió Catalana d'Hospitals. The patients/participants provided their written informed consent to participate in this study.

## Author Contributions

AP-L, DB-F, and JT-M participated in the initial conception of the design of the BBHI project. MR-C, DB-F, AP-L, GC, and RP-A contributed to conception and design of the present study. MR-C, SD-G, GE-I, VA-S, CP-G, SA, JS-S, and TM contributed to the acquisition of data. MR-C, GC, TM, HZ, and RP-A analyzed the data. MR-C, GC, and DB-F contributed to the draft of the manuscript. All authors contributed to manuscript revision, read, and approved the submitted version.

## Funding

DB-F and RP-A were funded by a Spanish Ministry of Science, Innovation and Universities (MICIU/FEDER; RTI2018-095181-B-C21) and an ICREA Academia 2019 Research Grants, and also supported by an ICREA Academia 2019 Grand Award, RP-A was supported by a fellowship from la Caixa Foundation (ID 100010434, Fellowship code: LCF/BQ/DI19/11730050), JT-M was partly supported Fundació Joan Ribas (Araquistain_FJRA), AGAUR, Agència de Gestió d'Ajuts Universitaris i de Recerca. Convocatòria 2018 d'Indústria del Coneixement (modalitat PRODUCTE) and FEDER, Fons Europeu de Desenvolupament Regional (2018 PROD 00172), Fundació La Marató De TV3 (201735.10), and European Commission - H2020/Call H2020-SC1-2016-2017 (RIA) (Grant Agreement No. 777107), AP-L was partly supported by the National Institutes of Health (R24AG06142, and P01 AG031720), the National Science Foundation, DARPA, and the Barcelona Brain Health Initiative funded primarily by La Caixa (LCF/PR/PR16/11110004), and HZ is a Wallenberg Scholar supported by grants from the Swedish Research Council (#2018-02532), the European Research Council (#681712), Swedish State Support for Clinical Research (#ALFGBG-720931), the Alzheimer Drug Discovery Foundation (ADDF), USA (#201809-2016862), the AD Strategic Fund and the Alzheimer's Association (#ADSF-21-831376-C, #ADSF-21-831381-C and #ADSF-21-831377-C), the Olav Thon Foundation, the Erling-Persson Family Foundation, Stiftelsen för Gamla Tjänarinnor, Hjärnfonden, Sweden (#FO2019-0228), the European Union's Horizon 2020 Research and Innovation Programme under the Marie Skłodowska-Curie grant agreement No 860197 (MIRIADE), and the UK Dementia Research Institute at UCL.

## Conflict of Interest

AP-L is a co-founder of Linus Health and TI Solutions AG; serves on the scientific advisory boards for Starlab Neuroscience, Magstim Inc., Radiant Hearts, and MedRhythms; is listed as an inventor on several issued and pending patents on the real-time integration of non-invasive brain stimulation with electroencephalography and magnetic resonance imaging and is an Associate Editor for Annals of Neurology. HZ has served at scientific advisory boards and/or as a consultant for Abbvie, Alector, Annexon, Artery Therapeutics, AZTherapies, CogRx, Denali, Eisai, Nervgen, Pinteon Therapeutics, Red Abbey Labs, Passage Bio, Roche, Samumed, Siemens Healthineers, Triplet Therapeutics, and Wave, has given lectures in symposia sponsored by Cellectricon, Fujirebio, Alzecure and Biogen, and is a co-founder of Brain Biomarker Solutions in Gothenburg AB (BBS), which is a part of the GU Ventures Incubator Program (outside submitted work). The remaining authors declare that the research was conducted in the absence of any commercial or financial relationships that could be construed as a potential conflict of interest.

## Publisher's Note

All claims expressed in this article are solely those of the authors and do not necessarily represent those of their affiliated organizations, or those of the publisher, the editors and the reviewers. Any product that may be evaluated in this article, or claim that may be made by its manufacturer, is not guaranteed or endorsed by the publisher.
