# Supplementary material for: Local Prefrontal Cortex TMS-Induced Reactivity Is Related to Working Memory and Reasoning in Middle-Aged Adults
Source: Front Psychol. 2022 Feb 10;13:813444. doi: 10.3389/fpsyg.2022.813444 (PMC8866698; doi:10.3389/fpsyg.2022.813444)
Supplement: Supplementary file 1 [file Data_Sheet_1.pdf]

# *Supplementary Material*

## 1 Targeting procedure

The cohort had two different target procedures (anatomical or parcellation method) because of other objectives in the main BBHI study. In order to control the possible effect of this variable on the predictors and outcome relationship, the target method was used as a covariate in the statistical analysis.

Twenty participants were included for the anatomical method, and targets were visually and anatomically defined by one investigator (TM). L-PFC stimulation was identified at the superior half of the middle frontal gyrus, approximately 3cm anterior to the precentral sulcus. L-IPL was selected at the superior edge of the angular gyrus around to intraparietal sulcus.

On the other hand, targets of 32 participants were determined based on a group-level resting-state 7 functional networks parcellation (Yeo et al., 2011). We followed the same methodology specified in Ozdemir et al., 2020 for this target procedure (Ozdemir et al., 2020). For each resting-state network, confidence maps were used, representing the confidence of each vertex belonging to its assigned network across a sample of 1000 healthy subjects (expressed as valued between −1 and 1), with larger values indicating high confidence. We used group-level functional parcellations and confidence maps on the Montreal Neurological Institute (MNI) template. We target the most consistent and reliable brain areas within the angular gyrus and the middle frontal gyrus, with the highest likelihood of occurring in the default mode network.

The mean coordinates of the anatomical targeting method were x=-33, y=37, z=52 for PFC, and x=-39, y=-69, z=53 for IPL. Whereas for the functional target method were x= -51, y= 30, z=33 for PFC, and x= -55, y= -69, z=30 for IPL.

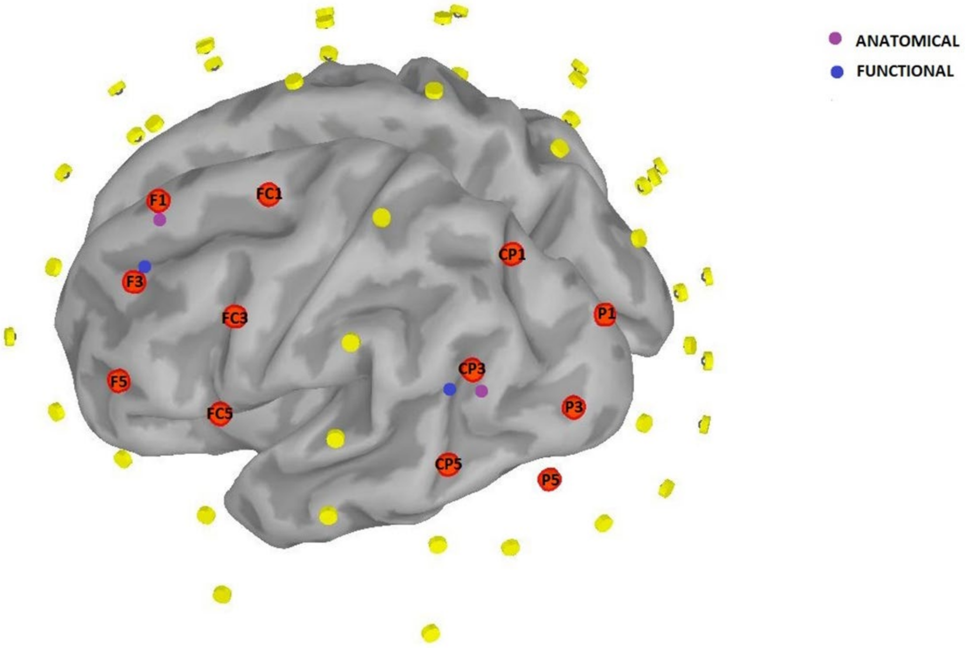


**Figure S1**: Mean L-PFC and L-IPL coordinates for the anatomical and functional targeting method. Red circles are the subset of electrodes used to calculate Local Mean-Field Power (LMFP) for each brain region. Both yellow and red circles are the electrodes used to calculate Global-Mean Field Power.

(A)

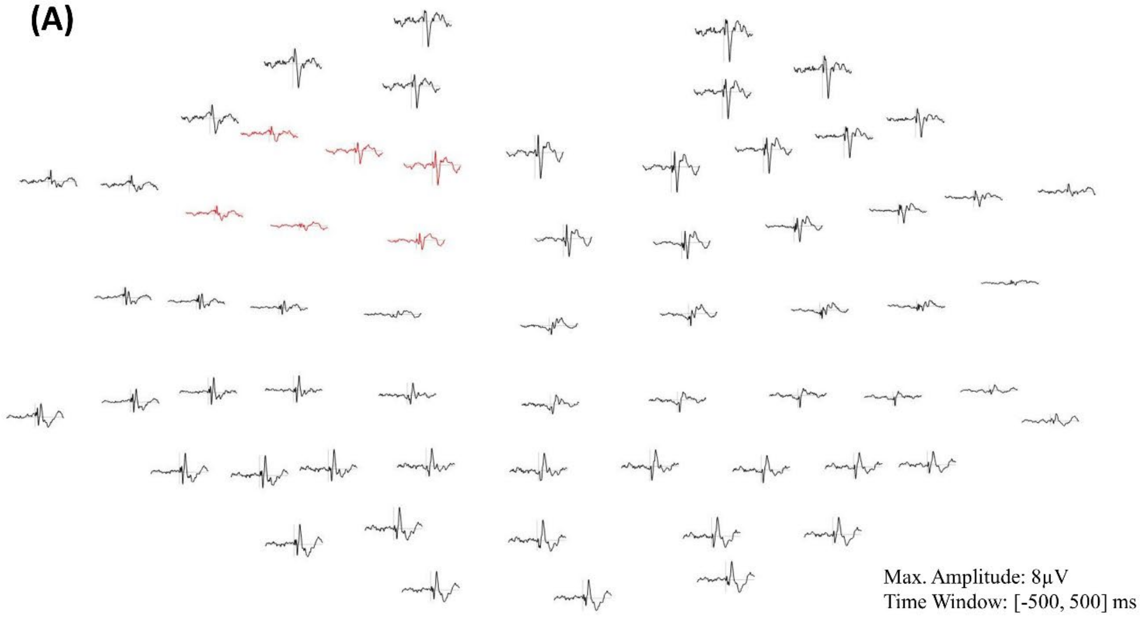


(B)

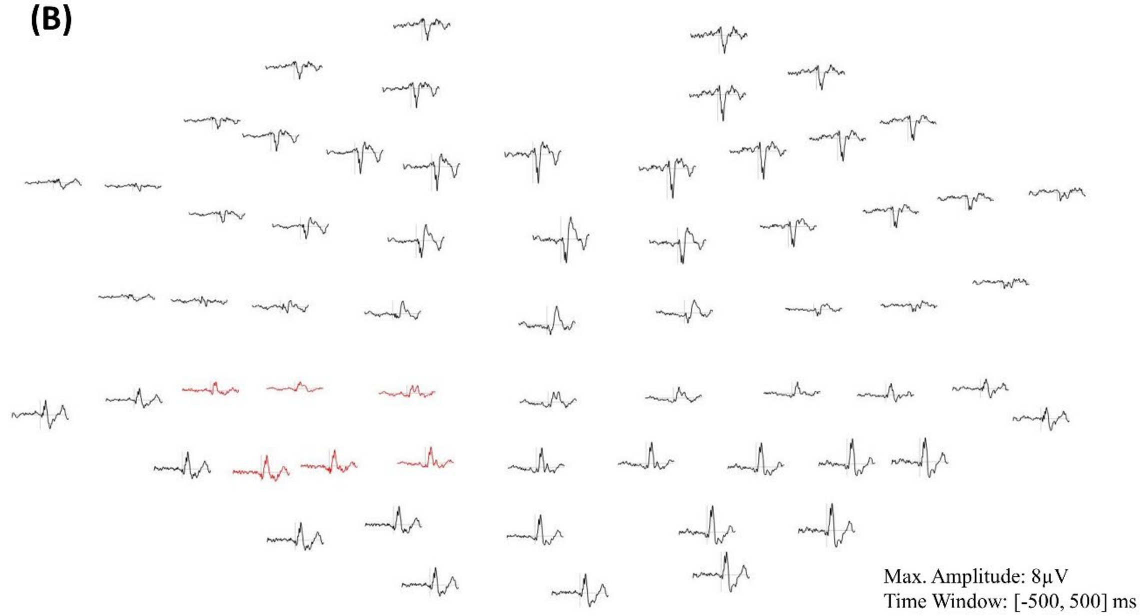


**Figure S2**: Average electroencephalographic responses from -500ms (pre-TMS) to +500ms (post-TMS) recorded from all EEG channels after PFC (A) and IPL (B) stimulation. Red responses correspond to the electrodes used to calculate LMFP.

# 2 Results

## 2.1 Descriptive statistics table

**Table S1**. Cognitive test scores divided into cognitive domains and functions (n=52)

| Cognitive Domain | Specific Cognitive Function | Cognitive Task | Min | Max | Mean | SD |
|---|---|---|---|---|---|---|
| Episodic Memory | Short-term memory | RAVLT immediate recall | 32 | 66 | 51.69 | 7.70 |
| | | Digit-Span Forward | 7 | 18 | 10.94 | 2.79 |
| | | Corsi block tapping | 8 | 19 | 14.65 | 2.73 |
| | Long-term memory | RAVLT delayed recall | 6 | 15 | 11.48 | 2.45 |
| | | RAVLT Recognition | 12 | 15 | 14.56 | .85 |
| Working Memory | Verbal working memory | Digit-Span Backward | 7 | 19 | 11.35 | 2.81 |
| | | Letter-Number Sequencing | 9 | 18 | 14.33 | 2.47 |
| Reasoning | Visuospatial reasoning | Matrix Reasoning WAIS-IV | 8 | 19 | 14.64 | 2.38 |
| | | Block Design WAIS-IV | 4 | 18 | 12.29 | 3.06 |
| Flexibility | Alternating attention | TMT B | 6 | 14 | 8.68 | 1.98 |
| Processing speed | Visuomotor speed | Digit symbol task | 9 | 19 | 13.71 | 2.55 |
| | Visual searching | TMT A | 6 | 18 | 11.21 | 2.80 |
| | Visual searching and selective attention | Cancellation test | 4 | 16 | 11.44 | 2.42 |

*Note:* RAVLT immediate recall= Recall a list of words immediately after hearing it of Rey Auditory Verbal Learning Test; RAVLT delayed recall = RAVLT recall after 30min; RAVLT recognition = Recognition of words from a word list of RAVLT; Digit-Span Forward = Immediate recall a series of numbers in the same order; Letter-Number Sequencing = Sequence a random order of numbers and letter; Matrix Reasoning WAIS-IV = Logical sequences and

series of Wechsler Adult Intelligence Scale-IV; Block Design WAIS-IV = Block Design of Wechsler Adult Intelligence Scale-IV; TMT B = Trail Making Test part B; TMT A = Trail Making Test part A; Digit symbol task = Digit symbol association; Cancellation test = cancellation task. All punctuations presented in this table are normalized scores except RAVLT tests that are raw scores.

## 2.2 Multivariate regression models for L-PFC and cognition (with covariates)

**Table S2.** Multivariate tests (PFC) including covariates.

| Effect | Value[1] | F | Hypothesis df | Error df | Sig. | Partial Eta Squared |
|---|---|---|---|---|---|---|
| Age | .631 | 3.28 | 5 | 28 | .019* | .369 |
| Biological sex | .732 | 2.05 | 5 | 28 | .102 | .268 |
| Education | .763 | 1.74 | 5 | 28 | .157 | .237 |
| Targeting method | .848 | 1.01 | 5 | 28 | .434 | .152 |
| NfL | .717 | 2.21 | 5 | 28 | .081 | .283 |
| LMFP Post-TMS | .658 | 2.91 | 5 | 28 | .031* | .342 |
| GMFP Post-TMS | .799 | 1.41 | 5 | 28 | .253 | .201 |

[1] Wilks' Lambda

*$p$<.05

**Table S3**. Tests of between subjects effects (PFC) including covariates.

| Source | Dependent Variable | Type III sum of squares | df | Mean Square | F | Sig. | Partial Eta Square |
|---|---|---|---|---|---|---|---|
| Targeting Method | Episodic Memory | .49 | 1 | .49 | .05 | .825 | .002 |
| | Reasoning | 5.21 | 1 | 5.21 | 3.17 | .084 | .090 |
| | Working Memory | 1.14 | 1 | 1.14 | .59 | .446 | .018 |

| | | | | | | | |
|---|---|---|---|---|---|---|---|
| | Processing speed | 1.51 | 1 | 1.51 | .40 | .531 | .012 |
| | Flexibility | .29 | 1 | .29 | .07 | .787 | .002 |
| Age | Episodic Memory | 8.93 | 1 | 8.93 | .90 | .349 | .027 |
| | Reasoning | 11.10 | 1 | 11.10 | 6.76 | .014 | .174 |
| | Working Memory | 4.73 | 1 | 4.73 | 2.46 | .127 | .071 |
| | Processing speed | 14.46 | 1 | 14.46 | 3.85 | .059 | .107 |
| | Flexibility | .93 | 1 | .93 | .24 | .628 | .007 |
| Biological sex | Episodic Memory | 8.34 | 1 | 8.34 | .84 | .366 | .026 |
| | Reasoning | 9.01 | 1 | 9.01 | 5.48 | .026 | .146 |
| | Working Memory | .15 | 1 | .15 | .08 | .779 | .002 |
| | Processing speed | 6.86 | 1 | 6.86 | 1.82 | .186 | .054 |
| | Flexibility | .88 | 1 | .88 | .23 | .637 | .007 |
| Education | Episodic Memory | 6.06 | 1 | 6.06 | .61 | .440 | .019 |
| | Reasoning | .64 | 1 | .64 | .39 | .536 | .012 |
| | Working Memory | 8.13 | 1 | 8.13 | 4.22 | .048 | .117 |
| | Processing speed | .27 | 1 | .27 | .07 | .792 | .002 |
| | Flexibility | 5.32 | 1 | 5.32 | 1.37 | .250 | .041 |
| NfL | Episodic Memory | .44 | 1 | .44 | .05 | .834 | .001 |
| | Reasoning | 5.09 | 1 | 5.09 | 3.10 | .088 | .088 |
| | Working Memory | 5.54 | 1 | 5.54 | 2.88 | .099 | .083 |
| | Processing speed | 8.09 | 1 | 8.09 | 2.15 | .152 | .063 |
| | Flexibility | 3.99 | 1 | 3.99 | 1.03 | .318 | .031 |
| GMFP Post-TMS | Episodic Memory | 2.84 | 1 | 2.84 | .29 | .596 | .009 |
| | Reasoning | 1.38 | 1 | 1.38 | .84 | .366 | .026 |

| | | | | | | | |
|---|---|---|---|---|---|---|---|
| | Working Memory | 2.29 | 1 | 2.29 | 1.19 | .283 | .036 |
| | Processing speed | 2.02 | 1 | 2.02 | .54 | .469 | .017 |
| | Flexibility | 9.27 | 1 | 9.27 | 2.39 | .132 | .070 |
| Local Post-TMS | Episodic Memory | .87 | 1 | .87 | .09 | .769 | .003 |
| | Reasoning | 7.73 | 1 | 7.73 | 4.70 | .038 | .128 |
| | Working Memory | 9.64 | 1 | 9.64 | 5.01 | .032 | .135 |
| | Processing speed | 9.93 | 1 | 9.93 | 2.64 | .114 | .076 |
| | Flexibility | 9.75 | 1 | 9.75 | 2.51 | .123 | .073 |

### 2.3 Multivariate regression models for L-PFC and cognition (without covariates)

**Table S4.** Multivariate tests (PFC) without covariates.

| Effect | Value[1] | F | Hypothesis df | Error df | Sig. | Partial Eta Squared |
|---|---|---|---|---|---|---|
| LMFP Post-TMS | .91 | .63 | 5 | 33 | .675 | .088 |
| GMFP Post-TMS | .82 | 1.45 | 5 | 33 | .232 | .182 |

[1] Wilks' Lambda

*$p$<.05

**Table S5**. Tests of between subjects effects (PFC) without covariates.

| Source | Dependent Variable | Type III sum of squares | df | Mean Square | F | Sig. | Partial Eta Square |
|---|---|---|---|---|---|---|---|
| GMFP Post-TMS | Episodic Memory | .75 | 1 | .75 | .08 | .777 | .002 |
| | Reasoning | .15 | 1 | .15 | .05 | .819 | .001 |

| | | | | | | | |
|---|---|---|---|---|---|---|---|
| | Working Memory | .03 | 1 | .03 | .01 | .908 | .000 |
| | Processing speed | 5.68 | 1 | 5.68 | 1.17 | .285 | .031 |
| | Flexibility | 8.57 | 1 | 8.57 | 2.25 | .142 | .057 |
| Local | Episodic Memory | 6.70 | 1 | 6.70 | .73 | .399 | .019 |
| Post-TMS | Reasoning | .16 | 1 | .16 | .06 | .813 | .002 |
| | Working Memory | 13.52 | 1 | 13.52 | 6.47 | .015 | .149 |
| | Processing speed | 12.36 | 1 | 12.36 | 2.56 | .118 | .065 |
| | Flexibility | 5.13 | 1 | 5.13 | 1.35 | .254 | .035 |

## 2.4 Multivariate regression models for L-IPL and cognition (with covariates)

**Table S6.** Multivariate tests (IPL) including covariates.

| **Effect** | **Value[1]** | **F** | **Hypothesis df** | **Error df** | **Sig.** | **Partial Eta Squared** |
|---|---|---|---|---|---|---|
| Age | .78 | 1.29 | 5 | 23 | .304 | .219 |
| Biological sex | .65 | 2.51 | 5 | 23 | .059 | .353 |
| Education | .92 | .39 | 5 | 23 | .850 | .078 |
| Targeting method | .66 | 2.37 | 5 | 23 | .072 | .340 |
| NfL | .86 | .74 | 5 | 23 | .601 | .139 |
| LMFP Post-TMS | .93 | .33 | 5 | 23 | .888 | .067 |
| GMFP Post-TMS | .91 | .44 | 5 | 23 | .815 | .088 |

[1] Wilks' Lambda

*$p$<.05

**Table S7**. Tests of between-subjects effects (IPL) including covariates.

| Source | Dependent Variable | Type III sum of squares | df | Mean Square | F | Sig. | Partial Eta Square |
|---|---|---|---|---|---|---|---|
| Targeting Method | Episodic Memory | .06 | 1 | .056 | .01 | .945 | .000 |
| | Reasoning | 14.90 | 1 | 14.896 | 6.34 | .018 | .190 |
| | Working Memory | .51 | 1 | .505 | .17 | .685 | .006 |
| | Processing speed | 1.27 | 1 | 1.268 | .30 | .592 | .011 |
| | Flexibility | 5.29 | 1 | 5.286 | 1.28 | .268 | .045 |
| Age | Episodic Memory | 7.44 | 1 | 7.440 | .65 | .428 | .023 |
| | Reasoning | 12.20 | 1 | 12.196 | 5.19 | .031 | .161 |
| | Working Memory | .03 | 1 | .026 | .01 | .927 | .000 |
| | Processing speed | 17.62 | 1 | 17.624 | 4.10 | .053 | .132 |
| | Flexibility | 1.71 | 1 | 1.713 | .41 | .525 | .015 |
| Biological sex | Episodic Memory | 1.26 | 1 | 1.260 | .11 | .743 | .004 |
| | Reasoning | 2.90 | 1 | 2.902 | 1.24 | .276 | .044 |
| | Working Memory | 11.34 | 1 | 11.340 | 3.77 | .063 | .122 |
| | Processing speed | .21 | 1 | .212 | .05 | .826 | .002 |
| | Flexibility | 1.45 | 1 | 1.449 | .35 | .559 | .013 |
| Education | Episodic Memory | 7.48 | 1 | 7.482 | .65 | .427 | .024 |
| | Reasoning | .31 | 1 | .311 | .13 | .719 | .005 |
| | Working Memory | 6.11 | 1 | 6.107 | 2.03 | .166 | .070 |
| | Processing speed | 2.54 | 1 | 2.540 | .59 | .449 | .021 |
| | Flexibility | 5.54 | 1 | 5.536 | 1.34 | .257 | .047 |
| NfL | Episodic Memory | 8.02 | 1 | 8.015 | .70 | .411 | .025 |

| | | | | | | | |
|---|---|---|---|---|---|---|---|
| | Reasoning | 1.02 | 1 | 1.021 | .43 | .516 | .016 |
| | Working Memory | 2.23 | 1 | 2.228 | .74 | .397 | .027 |
| | Processing speed | 5.22 | 1 | 5.221 | 1.21 | .280 | .043 |
| | Flexibility | .17 | 1 | .171 | .04 | .840 | .002 |
| GMFP Post-TMS | Episodic Memory | 2.01 | 1 | 2.008 | .18 | .679 | .006 |
| | Reasoning | 2.79 | 1 | 2.785 | 1.19 | .286 | .042 |
| | Working Memory | 1.35 | 1 | 1.351 | .45 | .509 | .016 |
| | Processing speed | .09 | 1 | .094 | .02 | .883 | .001 |
| | Flexibility | .45 | 1 | .446 | .11 | .745 | .004 |
| Local Post-TMS | Episodic Memory | 3.45 | 1 | 3.446 | .30 | .589 | .011 |
| | Reasoning | 1.96 | 1 | 1.957 | .83 | .370 | .030 |
| | Working Memory | .50 | 1 | .504 | .17 | .686 | .006 |
| | Processing speed | .03 | 1 | .032 | .01 | .932 | .000 |
| | Flexibility | .11 | 1 | .113 | .03 | .870 | .001 |

## 2.5 Multivariate regression models for L-IPL and cognition (without covariates)

**Table S8.** Multivariate tests (IPL) without covariates.

| **Effect** | **Value[1]** | **F** | **Hypothesis df** | **Error df** | **Sig.** | **Partial Eta Squared** |
|---|---|---|---|---|---|---|
| LMFP Post-TMS | .97 | .19 | 5 | 28 | .965 | .032 |
| GMFP Post-TMS | .94 | .39 | 5 | 28 | .854 | .064 |

[1] Wilks' Lambda

**p*<.05

**Table S9**. Tests of between subjects effects (IPL) without covariates.

| Source | Dependent Variable | Type III sum of squares | df | Mean Square | F | Sig. | Partial Eta Square |
|---|---|---|---|---|---|---|---|
| GMFP Post-TMS | Episodic Memory | .21 | 1 | .21 | .02 | .889 | .001 |
| | Reasoning | .15 | 1 | .15 | .05 | .828 | .002 |
| | Working Memory | 2.81 | 1 | 2.81 | .82 | .373 | .025 |
| | Processing speed | 1.00 | 1 | 1.00 | .19 | .670 | .006 |
| | Flexibility | .24 | 1 | .24 | .06 | .808 | .002 |
| Local Post-TMS | Episodic Memory | 1.07 | 1 | 1.07 | .10 | .749 | .003 |
| | Reasoning | .01 | 1 | .01 | .00 | .957 | .000 |
| | Working Memory | 1.31 | 1 | 1.31 | .38 | .542 | .012 |
| | Processing speed | .27 | 1 | .27 | .05 | .824 | .002 |
| | Flexibility | .29 | 1 | .29 | .07 | .788 | .002 |

## 2.6 Neurofilaments level and cognition correlations

**Table S10.** NfL level and cognitive functions

| | NfL (*r*) |
|---|---|
| Episodic Memory | .050 |
| Working Memory | .046 |
| Reasoning | -.373** |
| Processing Speed | -.338* |
| Flexibility | -.293* |

*$p$<.05. **$p$<.01

## 3 Supplementary material references:

Yeo. B. T.. Krienen. F. M.. Sepulcre. J.. Sabuncu. M. R.. Lashkari. D.. Hollinshead. M.. et al. (2011). The organization of the human cerebral cortex estimated by intrinsic functional connectivity. *J. Neurophysiol.* 106. 1125–1165. doi:10.1152/jn.00338.2011.
